# Successful surgical treatment of extrahepatic biliary papillomatosis diagnosed with endoscopic retrograde cholangiopancreatography: a case report

**DOI:** 10.1186/1752-1947-8-148

**Published:** 2014-05-13

**Authors:** Tarik Adioui, Hassan Seddik, Hicham Baba, Badr Slioui, Abdelmounaim Ait Ali, Fatima Zohra El Hamdi, Ahmed Benkirane, Aziz Zentar

**Affiliations:** 1 Department of Gastroenterology II, Mohamed V Teaching Military Hospital, Rabat 10100, Morocco; 2 Department of General Surgery I, Mohamed V Teaching Military Hospital, Rabat 10100, Morocco; 3 Department of Radiology, Mohamed V Teaching Military Hospital, Rabat 10100, Morocco

**Keywords:** Biliary papillomatosis, Biliary tract, Obstructive jaundice

## Abstract

**Introduction:**

Biliary papillomatosis is a condition characterized by multiple papillary tumors of variable distribution and extent within the biliary tract. Papillary carcinoma can develop in these lesions. It is a rare biliary pathological entity and its clinical features and outcome are not well known.

**Case presentation:**

We experienced a case of biliary papillomatosis in a 51-year-old North African man who presented with obstructive jaundice. Laboratory tests showed elevated bilirubin, alkaline phosphatase and gamma-glutamyl transpeptidase levels. Imaging (ultrasound and magnetic resonance imaging) was suggestive of Klatskin tumor associated to common bile duct stones. After endoscopic retrograde cholangiopancreatography, a balloon sweep retrieved friable tissue from his bile ducts. Histology demonstrated papillary adenomatous proliferation showing high-grade dysplasia and he was referred for surgical management.

**Conclusions:**

Although biliary papillomatosis is rare, it is a premalignant condition that should be well known and considered in all diagnoses of obstructive jaundice. We report a new case of biliary papillomatosis and highlight the contribution of endoscopic retrograde cholangiopancreatography in the diagnosis of this condition.

## Introduction

Biliary papillomatosis (BP) is a rare condition of unknown etiology characterized by multiple papillary tumors within the intrahepatic and/or extrahepatic biliary tree that can cause biliary obstruction [1]. It is a low-grade neoplasm with high malignant potential and should be regarded as a premalignant lesion. BP commonly affects adults in their sixth to seventh decade of life and is twice as common in males as females [1,2]. It presents with recurrent episodes of abdominal colic, jaundice, acute cholangitis and the presence of biliary stones and/or infection. We describe a case of BP in which endoscopic retrograde cholangiopancreatography (ERCP) played an important role in the diagnosis of this condition.

## Case presentation

**Figure 1:**
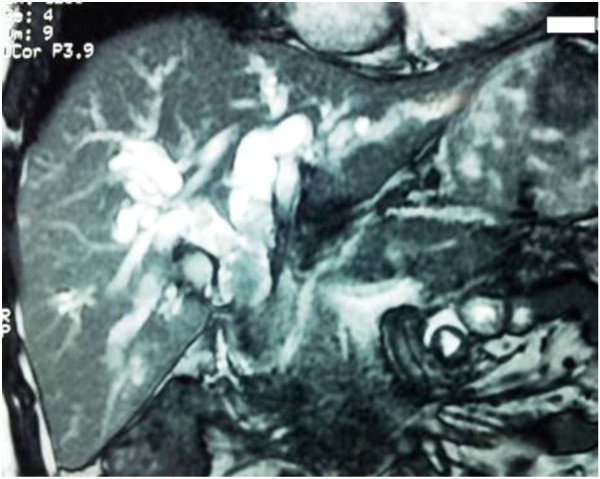
Magnetic resonance image showing a complete stop at the biliary bifurcation compatible with a Klatskin tumor associated to common bile duct stones.

**Figure 2:**
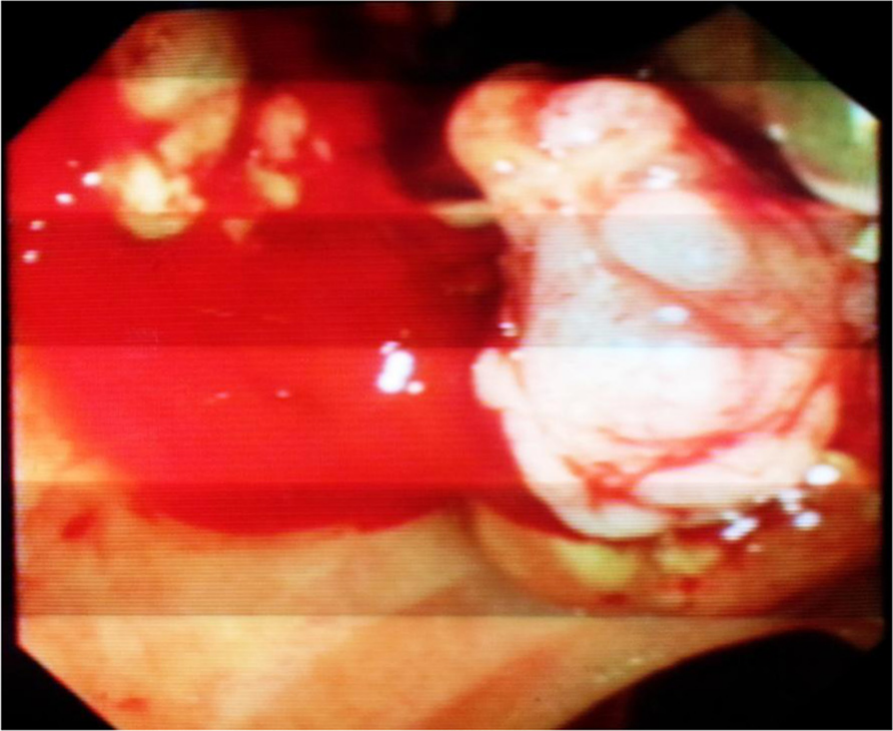
After endoscopic retrograde cholangiopancreatography, balloon sweep retrieved friable, polypoid soft tissue from bile ducts.

**Figure 3:**
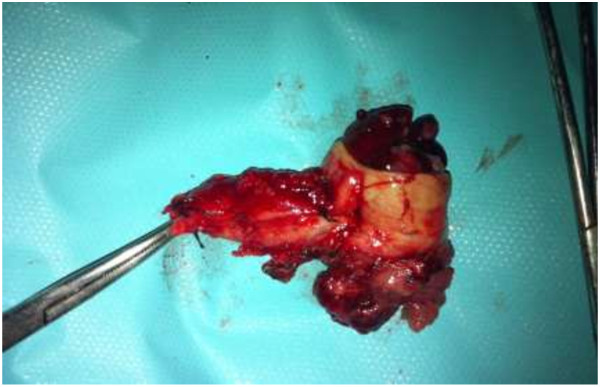
Post operative view of common bile duct containing papillary material.

**Figure 4:**
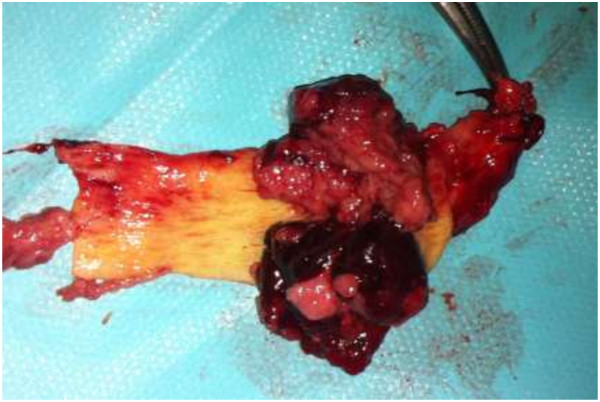
Surgical specimen showing polylobed papillary mass within the common bile duct.

A 51-year-old North African man presented with a 1-month history of fluctuating jaundice with pale stools and dark urine, right upper quadrant pain, and intermittent vomiting. His past medical history included neurosyphilis for approximately 8 years. A physical examination revealed little upper abdominal quadrant tenderness. Biochemistry demonstrated elevated bilirubin, alkaline phosphatase and γ-glutamyl transpeptidase levels, and leukocytosis. Ultrasound and magnetic resonance imaging showed a complete stop at the biliary bifurcation compatible with a Klatskin tumor associated to common bile duct stones (Figure 1). Endoscopic retrograde cholangiography revealed dilated intrahepatic ducts and common bile duct above multiple polylobed filling defects. These findings were mainly suggestive of multiple impacted stones of his common bile duct, cholangiocarcinoma, or hemobilia. After an endoscopic sphincterotomy, a balloon sweep retrieved friable, polypoid soft tissue from his bile ducts (Figure 2). Two biliary plastic stents were placed. Histology of the tissue demonstrated papillary adenomatous proliferation showing high-grade dysplasia; the patient was referred for surgical management. Laparoscopic exploration demonstrated a dilated common biliary duct. A transversal choledochotomy revealed a papillary polylobed mass (Figures 3 and 4). Common bile duct resection was performed with macroscopically safe margins and biliary reconstruction was achieved by Roux-en-Y hepaticojejunostomy.

## Discussion

BP is papillary adenomatosis within the biliary tree. It is a very rare disorder that typically presents with fluctuant obstructive jaundice.

BP can be thought of as a collection of benign papillary adenomas within which adenocarcinoma can develop and spread along the bile duct mucosa [1-3]. Recent literature suggests BP has a rate of malignant transformation between 41%, and 83% [1,2].

Since the first case described by Chappet in 1894, almost 100 cases have been reported.

BP pathogenesis is unclear and appears related to irritation and inflammation of the biliary tree. The roles of bile stasis and recurrent infections are also underlined [4]. Some associations were described: chronic stone irritation, *clonorchis* infestation, reactive hyperplasia in Caroli’s disease, ectopic pancreatic tissue, anomalous biliary tree and primitive sclerosing cholangitis [5]. Our patient had neurosyphilis and, to the best of knowledge, this is the first report of this association.

Clinical features are obstructive jaundice and recurrent cholangitic episodes. These features are due to partial or intermittent obstruction of the bile duct secondary to mucus production, tumor enlargement or tumor fragments [6,7].

The most common imaging finding is intrahepatic ducts and/or common bile duct dilatation (polypoid filling defects, mural irregularities) [8]. Other features such as the presence of multiple intraductal masses within the lumen or attached to the wall of bile ducts are typically visualized on magnetic resonance cholangiopancreatography [2-6]. Computed tomography scan may reveal soft tissue densities in the dilated bile ducts or thickened and enhanced bile duct walls [1]. During ERCP, typical characteristics include: amorphous filling defects, irregularity of the bile duct wall, reduced motility on irrigation and mucus discharge from the papilla. BP may then be classified as either mucin-hypersecreting BP or non-mucin-producing BP depending on the presence or absence of mucobilia. These morphological features are mainly suggestive of hilar cholangiocarcinoma, and we aim here to draw readers’ attention to consider BP in all diagnosis of hilar tumors, which avoids condemning patients with palliative therapy systematically. During a contrast cholangiogram, it is important to obtain opacification of the entire biliary tract to determine precisely the ductal extension of the disease, which helps to guide the treatment plan, including surgery [9,10].

Curative treatment can be achieved through surgical resection in localized disease [2-11]. However, local resection is characterized by a high rate of recurrence due to either positive resection margins or recurrence due to the multifocality of the disease. In the case of diffuse or recurrent BP, resection of the biliary tree by liver transplantation and pancreaticoduodenectomy can be curative [1,2,12,13].

Endoscopic therapy through ERCP was initially used for palliation in patients who were poor surgical candidates. Argon plasma coagulation has been attempted for the treatment of premalignant mucosal diseases of the digestive tract, due to its low depth and satisfactory local effects, and its use in the biliary tree has been scarcely described [14]. Palliative procedures also include percutaneous management with drainage and stenting, and intraluminal brachytherapy with iridium-192. Untreated BP that does not undergo malignant transformation eventually leads to chronic cholestasis due to mechanical obstruction resulting in septic cholangitis and hepatic failure [2].

## Conclusions

BP is a rare condition characterized by multiple papillary adenomas involving the biliary tract which leads to recurrent cholangitis. It is considered a rare benign disease with a high malignancy potential and tendency to spread superficially. Definitive treatment is by means of radical surgical resection and/or liver transplantation.

## Consent

Written informed consent was obtained from the patient for publication of this case report and accompanying images. A copy of the written consent is available for review by the Editor-in-Chief of this journal.

## Abbreviations

BP: Biliary papillomatosis; ERCP: Endoscopic retrograde cholangiopancreatography.

## Competing interests

The authors declare that they have no competing interests.

## Authors’ contributions

AT and SH evaluated the patient and were major contributors in writing the manuscript. SB analyzed MRI data. SH and FZE realized the ERCP. BH, AA, and ZA performed the surgical management. BA and ZA reviewed the manuscript. All authors read and approved the final manuscript.
